# Inhibition of BACE1 attenuates microglia-induced neuroinflammation after intracerebral hemorrhage by suppressing STAT3 activation

**DOI:** 10.18632/aging.204935

**Published:** 2023-08-07

**Authors:** Jianfeng Zhuang, Yang Cao, Gengyin Guo, Maogui Li, Tongfu Zhang, Dong He, Jinyan Chen, Keke Zhang, Zhen Zhang

**Affiliations:** 1 Department of Neurosurgery, Qilu Hospital, Shandong University, Jinan 250012, China; 2 Department of Neurosurgery, Affiliated Hangzhou First People’s Hospital, Zhejiang University School of Medicine, Hangzhou 310003, China; 3 Department of Neurosurgery, Shandong Provincial Hospital Affiliated to Shandong First Medical University, Jinan 250021, China

**Keywords:** intracerebral hemorrhage, neuroinflammation, BACE1, STAT3, microglia

## Abstract

Hematoma-induced neuroinflammation is the cause of poor prognosis in intracerebral hemorrhage (ICH); therefore, promoting blood clearance and blocking overactivated inflammation are rational approaches for ICH treatment. β-site amyloid precursor protein (APP) lyase-1 (BACE1) is a key molecule regulating the microglial phenotype transition in neurodegenerative diseases. Therefore, the aim of this study was to investigate the role of BACE1 in microglial phagocytosis and inflammatory features in ICH. Here, we demonstrated the unique advantages of targeting BACE1 in microglia using an autologous blood model and primary microglia hemoglobin stimulation. When BACE1 was inhibited early in ICH, fewer residual hematomas remained, consistent with an increase in genetic features that favor phagocytosis and anti-inflammation. In addition, inhibition of BACE1 enhanced the secretion of anti-inflammatory cytokines and substantially reduced the expression of proinflammatory genes, which was regulated by signal transduction and phosphorylation of activator of transcription 3 (STAT3). Further pharmacological inhibition of STAT3 phosphorylation effectively blocked the proinflammatory and weak phagocytic phenotype of microglia due to BACE1 induction. In summary, BACE1 is the critical molecule regulating the inflammatory and phagocytic phenotypes of microglia after ICH, and targeted inhibition of the BACE1/STAT3 pathway is an important strategy for the future treatment of ICH-induced neurological injury.

## INTRODUCTION

Intracerebral hemorrhage (ICH) is a devastating cerebrovascular disease with high mortality and morbidity worldwide that lacks specific treatment. The global burden of ICH continues to increase year by year, with increasing prevalence in younger age populations [1, 2]. To develop an effective treatment strategy for ICH, researchers require an in-depth understanding of its pathophysiology. Recently, the pathological process of ICH was shown to involve hematoma formation-induced mechanical mass, which indicates primary brain damage, and the lysis of erythrocytes leading to delayed secondary brain injury occurring in the perihematomal area [3].

Neuroinflammation is a key pathological mechanism throughout secondary brain injury, mainly manifested in the activation of microglia, which are the most important type of innate immune cells in the central nervous system (CNS) and the first responders to ICH injury [4, 5]. Microglia exhibits a high degree of plasticity and diverse functions throughout the course of injury that are relevant to disease progression and regression [6–8]. Appropriate modulation of the excessive inflammatory response of microglia contributes to the secretion of anti-inflammatory factors and the production of pro-repair molecules, as well as to accelerated erythrocyte clearance, acceleration of vascular regeneration and recovery of white matter damage [9]. As a vital regulator of microglia, BACE1 is elevated in cognitive impairment-associated inflammatory microglia, whereas there is a concomitant decrease in phagocytosis and immunosurveillance [10]. Although the biological functions of BACE1 in microglia after ICH remain unclear, the correlation between BACE1 elevation and LPS-induced inflammation in a microglial cell model suggests that BACE1 may participate in ICH secondary brain injury [11].

In recent studies, inhibition of microglial BACE1, which regulates microglial gene signatures associated with phagocytosis, has been suggested as a superior strategy for Alzheimer’s disease [12]. Moreover, BACE1 is upregulated in the hippocampus induced by systemic inflammation [13]. MK-8931 (Verubecestat), a non-peptidic class of BACE1 inhibitor, which originally developed for AD treatment penetrates the blood-brain barrier (BBB) very well and blocks BACE1 activity efficiently in the brain [14, 15]. Therefore, MK-8931 can be considered as an effective tool to inhibit the function of BACE1 in brain [16]. In this study, we found that BACE1 was preferentially upregulated in microglia at the acute stage after ICH and that STAT3 signaling was required to maintain BACE1-induced inflammation and phagocytosis in microglia. We demonstrated that the BACE1 inhibitor MK-8931, which has been tested in clinical trials for AD treatment, could effectively converts proinflammatory microglia into an anti-inflammatory state and promotes phagocytosis of hematoma *in vitro* and *in vivo*. Collectively, this study shows that microglial BACE1 is a promising approach to promote functional recovery after ICH and elucidates the underlying mechanism of neuroinflammation and hematoma resolution.

## RESULTS

### Elevated BACE1 expression in microglia induced by ICH

**Figure 1:**
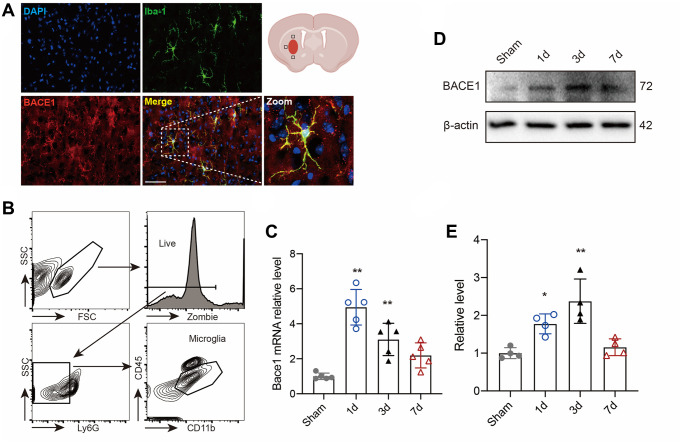
**BACE1 increased in microglia after ICH.** (**A**) Representative immunofluorescent images of BACE1 and the microglial marker IBA1 in 3-day ICH brain samples. (**B**) Gating strategy of microglia (Zombie-Ly6G-CD45intCD11b+) (CD45intCD11b+) sorting after ICH. (**C**) Time course of Bace1 gene expression in FACS-isolated microglia. (**D**) Representative blot images of BACE1 protein levels in FACS-isolated microglia. (**E**) Quantitative analysis of the relative change in BACE1 (percentage of sham group). Data are expressed as the means ± SDs; *n* = 54. ^*^P < 0.05, ^**^P < 0.01 vs. the sham group.

BACE1 protein levels and activity in microglia were shown to be increased in several neurological diseases [12, 17, 18]. Here, we investigated the location and expression of BACE1 in microglia following ICH-mediated acute injury. We found that activated microglia from mouse brains 3 d post-ICH showed strong colocalization of BACE1 with ionized calcium-binding adapter molecule 1 (IBA-1) (Figure 1A). We isolated microglia (CD45intCD11b+) in the striatum from mouse brains at different time points after ICH modeling (Figure 1B) and measured the temporal expression of microglial Bace1. The expression of BACE1 peaked rapidly at 1 day post-ICH and persisted until 3 days, while the mRNA level showed no significant difference with baseline at 7 days after ICH (Figure 1C). Consistently, Western blotting also indicated that the protein level of microglial BACE1 significantly increased at Day 1 and Day 3 post-ICH (Figure 1D, 1E). Hence, BACE1 was elevated in ICH-affected microglial cells at an early stage.

### BACE1 is involved in microglia-mediated neural injury

**Figure 2:**
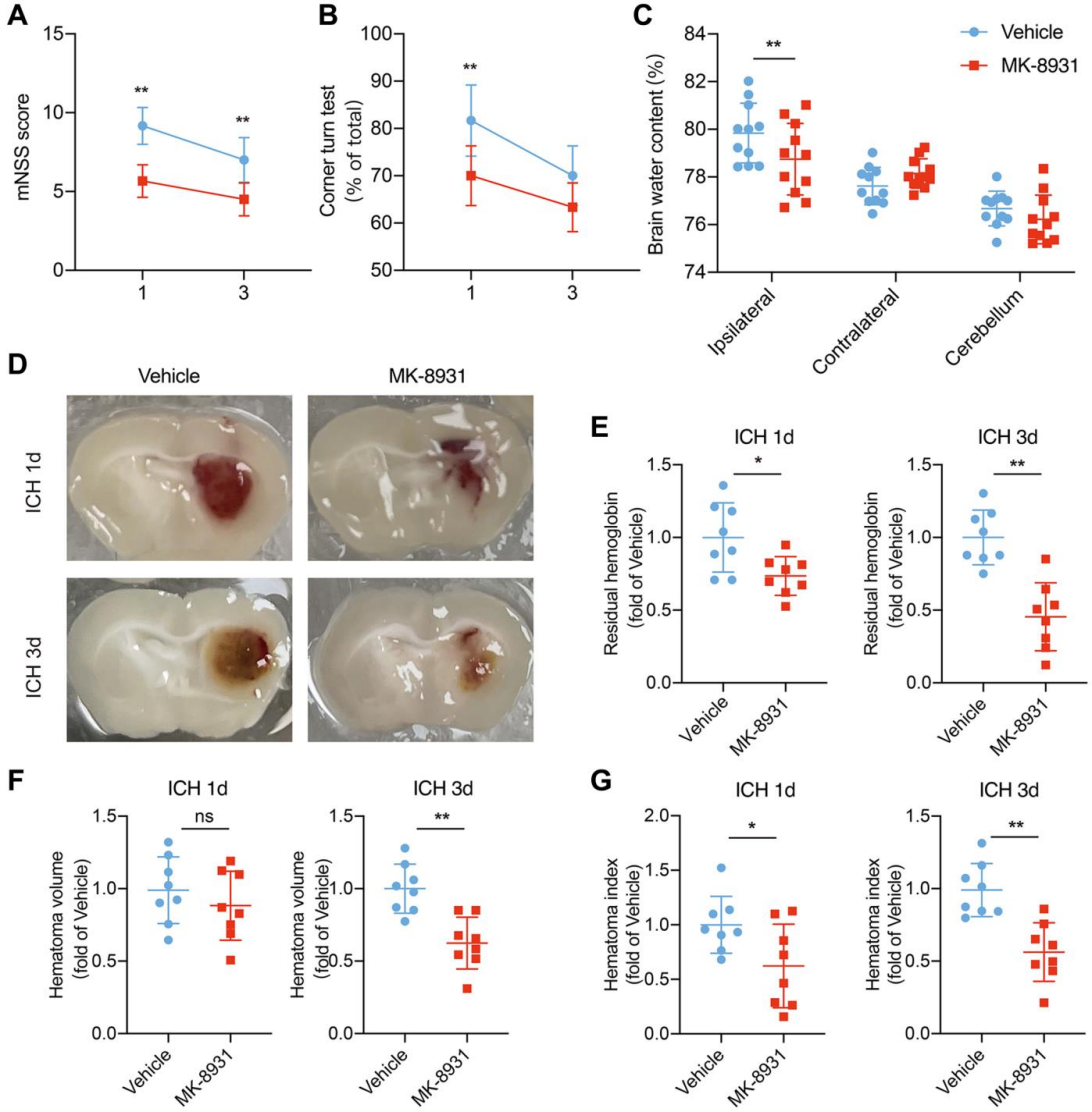
**MK-8931 attenuated ICH induced neurological deficits via BACE1 inhibition.** (**A**, **B**) ICH was induced in mice by injection of autologous blood. Summarized results showing neurological assessment (mNSS score, corner-turning test) of the MK-8931- or vehicle-treated groups of mice. *n* = 8 per group. (**C**) Brain water content in groups of mice receiving the indicated treatments at Day 1 after ICH. *n* = 11 per group. (**D**) Representative coronal sections showing hematomas from the indicated treated mice. (**E**–**G**) Quantification of residual hemoglobin, hematoma volume, and hematoma index (volume × density). *n* = 8 per group. Data are presented as the mean ± SD. ^*^P < 0.05, ^**^P < 0.01.

To explore the role of BACE1 upregulation in microglia, we used MK-8931, a BACE1-specific inhibitor, to block the function of BACE1 [10]. Given that microglia are essential to ICH recovery via the inflammatory response, brain edema and hematoma clearance [19, 20], we wondered whether BACE1 would affect the above microglial functions and neurological impairments. We first measured the mNSS score and corner turn rate in the mice with ICH that received MK-8931 or vehicle at 1 and 3 days after ICH induction. The inhibition of BACE1 significantly reduced neurological deficits (Figure 2A, 2B). Moreover, we found that the brain water content following ICH at 1 day was lower in the MK-8931-treated mice (Figure 2C). Next, we compared the differences in hematoma clearance based on a previous study. At 3 days post-ICH, MK-8931 dramatically reduced the residual hematoma volume and hemoglobin levels compared to the vehicle, and even as early as 1 day after ICH, the residual blood was significantly decreased in the MK-8931-treated mice, although the residual hematoma volume was similar to that of the control group (Figure 2D–2G). In conclusion, BACE1 might promote neuroinflammation and inhibit phagocytosis by microglia, thereby affecting recovery from ICH injury.

### BACE1 elevated neuroinflammation and impaired erythrophagocytosis of microglia

**Figure 3:**
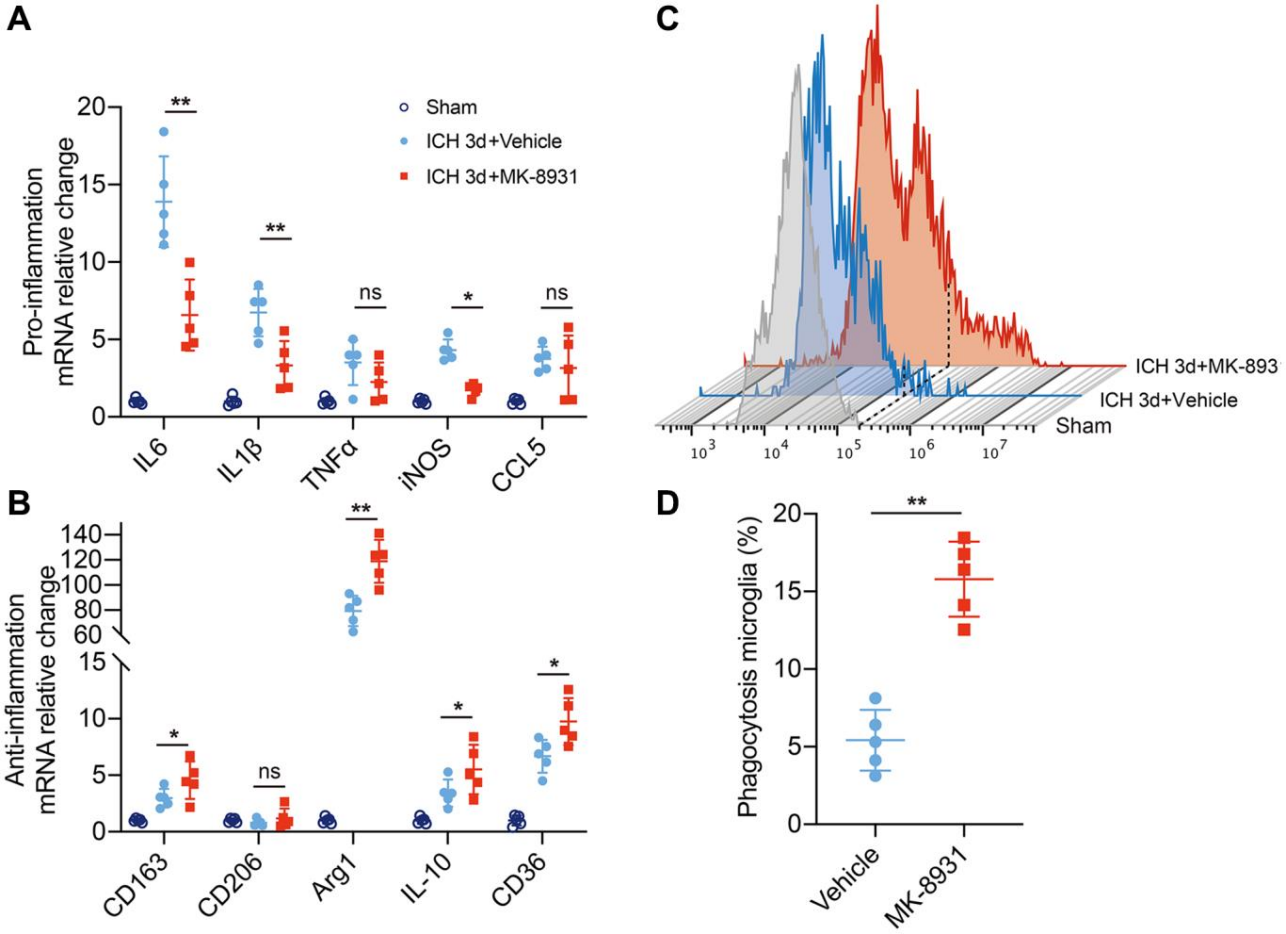
**BACE1 correlated with microglial neuroinflammation and erythrophagocytosis.** (**A**, **B**) Quantification of pro- or anti-inflammatory cytokine gene expression is shown in the bar graphs, with data from at least 5 mice at 3 days post-ICH. Data are shown as the relative change compared to the sham group, ^*^P < 0.05 and ^**^P < 0.01. (**C**) Flow cytometry histogram of fluorescently labeled erythrocytes in microglia/macrophages. (**D**) Quantification of the erythrophagocytosis population of microglia in the indicated treated mice at Day 3 after ICH. *n* = 5 per group. ^**^P < 0.01.

To explore the exact relationship between BACE1 and inflammation and phagocytosis, we isolated microglia in the striatum from ICH 3-day mouse brains and measured the levels of classical pro/anti-inflammatory cytokines. In the MK-8931-treated mice, the expression of the IL-6, IL-1β and iNOS genes was significantly decreased in sorted microglia beginning at 3 days post-ICH, whereas the anti-inflammatory factors Arg1 and IL-10 and the pro-hemoglobin/erythrocyte phagocytic molecules CD163 and CD36 were significantly upregulated (Figure 3A, 3B). We further used fluorescence-labeled RBCs to construct an autologous blood-brain hemorrhage model to confirm the phagocytic capacity of microglia. We found a higher percentage of microglia in the MK-8931-treated mice that underwent erythrophagocytosis than in the vehicle-treated mice (Figure 3C, 3D). Thus, inhibition of BACE1 promoted a shift from a proinflammatory phenotype to an anti-inflammatory, pro-clearing phenotype in microglia after cerebral hemorrhage.

### BACE1 was detrimental to the transformation of the microglial protective phenotype *in vitro*

**Figure 4:**
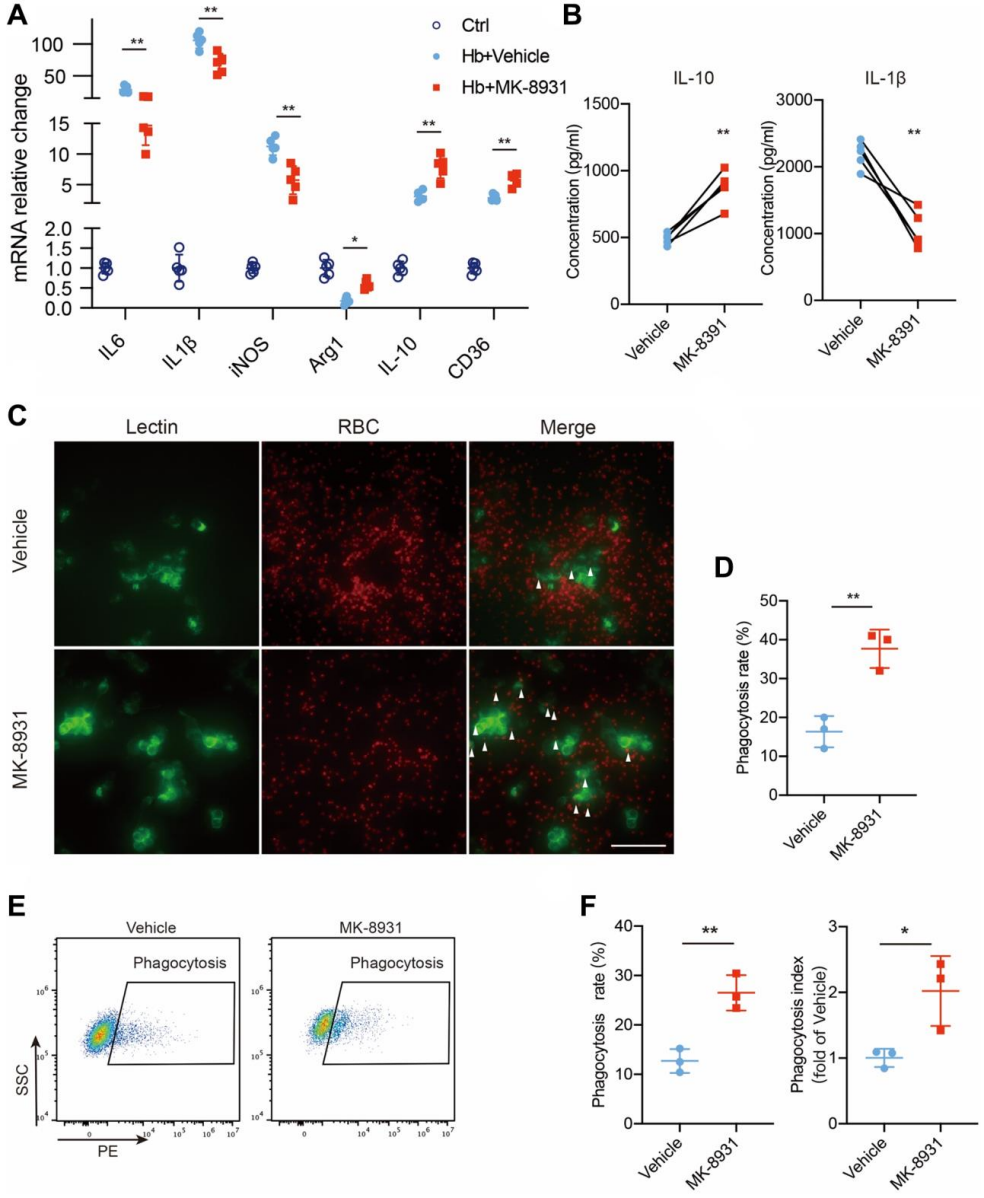
**BACE1 impaired protective phenotype transition of microglia *in vitro*.** (**A**) Quantification of cytokine gene expression is shown in the bar graphs, with data from at least 5 independent experiments. Data are shown as the relative change of the Ctrl group, ^*^P < 0.05 and ^**^P < 0.01. (**B**) Secreted IL-10 and IL-1β protein levels in the vehicle- and MK-8931-treated PMG measured by ELISAs. (**C**) Representative images of *in vitro* erythrophagocytosis of PMG, scale bar = 20 μm. (**D**) The percentage of erythrophagocytosis was calculated in the indicated treated PMG. (**E**, **F**) *In vitro* analysis of PMG erythrophagocytosis under Hb stimulation with/without MK-8901 treatment. The phagocytosis index was calculated as the median fluorescence intensity of the RBC-engulfed population, *n* = 3 per group. All data are presented as the mean ± SD. ^*^P < 0.05, ^**^P < 0.01.

To further determine the role of BACE1 in microglial inflammatory and phagocytic phenotypes, we used hemoglobin (Hb)-treated primary microglia (PMG) as an *in vitro* model of ICH [21]. The BACE1 protein level was increased at 6 and 12 h after Hb stimulation (Supplementary Figure 1). Then, we analyzed cytokine gene expression in the MK-8931- or vehicle-treated PMG. Consistent with the *in vivo* results, proinflammatory cytokines (IL-6, IL-1β, iNOS) were significantly reduced by MK-8931, while anti-inflammatory/pro-phagocytosis molecules were dramatically increased (Figure 4A), and the results also validated by BACE1-targeted siRNA (Supplementary Figure 2A, 2B). The secreted IL-10 levels also increased significantly after MK-8931 treatment, while the secreted IL-1β levels decreased significantly in the PMG (Figure 4B). Thus, inhibition of BACE1 contributes to the conversion of microglia to an anti-inflammatory phenotype. We further measured the phagocytosis rate of the Hb-stimulated PMG with or without BACE1 inhibition by immunofluorescence and flow cytometry. We found that RBCs accumulated around the PMG, suggesting insufficient phagocytosis. In contrast, the number of intracellular fluorescence-labeled RBCs was significantly increased after the administration of MK-8931, and the aggregation of RBCs around the PMG disappeared (Figure 4C, 4D). Quantitative analysis of the proportion of PMG that underwent phagocytosis and their fluorescence intensity (indicating that one cell may involve multiple RBCs) revealed that that pharmacological inhibition of BACE1 by MK-8901 significantly enhanced the phagocytosis of PMG (Figure 4E, 4F) as well as the genetic inhibition by siRNA (Supplementary Figure 2C, 2D). Together, our results indicated that BACE1 inhibition contributes to a greater tendency of microglia to exhibit anti-inflammatory and pro-phagocytic phenotypes.

### BACE1 mediates microglial phenotype by activating STAT3 signaling

**Figure 5:**
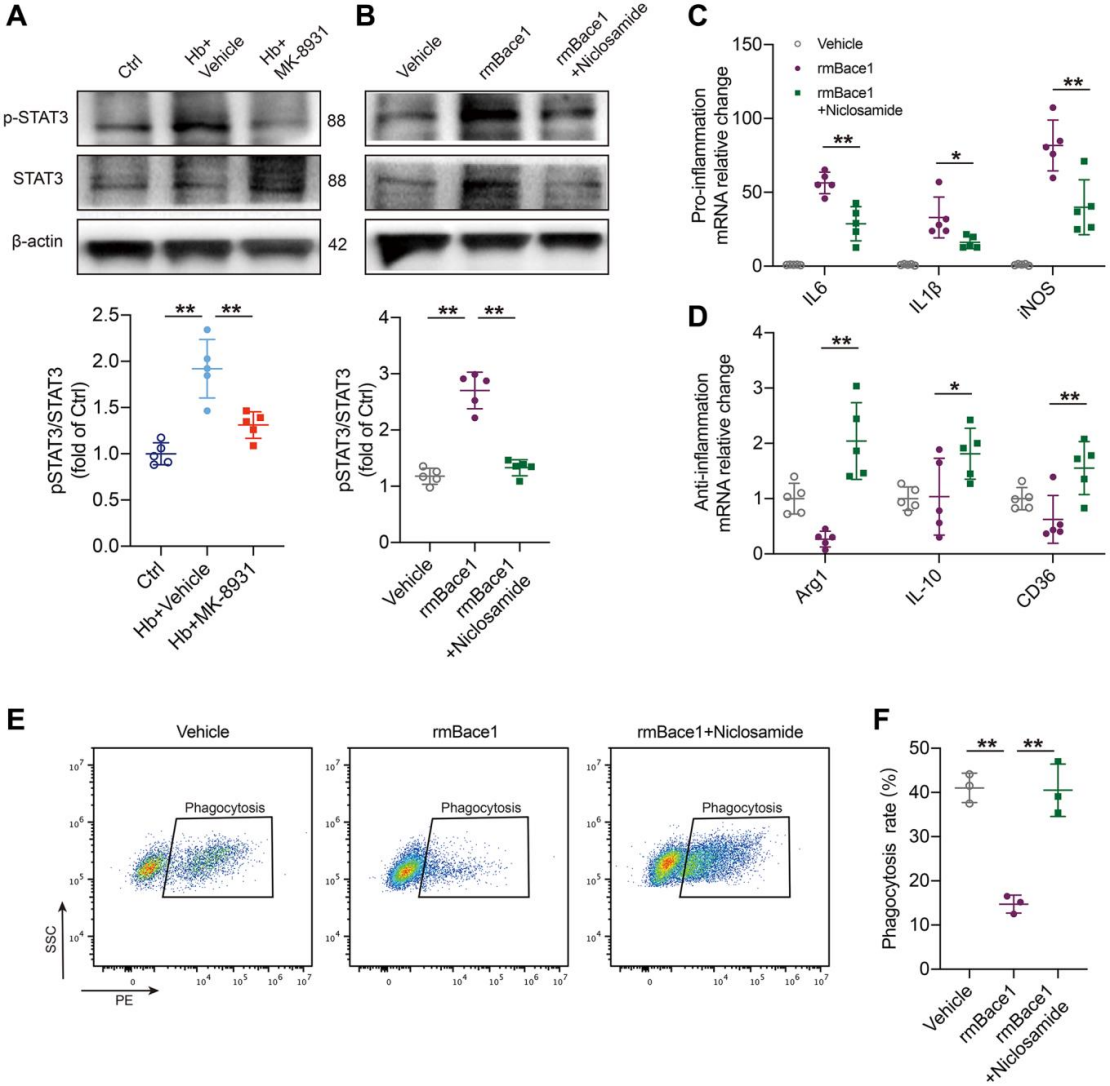
**BACE1 induced microglia pro-inflammatory phenotype by STAT3 signaling activation.** (**A**) The levels of phosphorylated and total STAT3 relative to the internal control actin are shown in the indicated treated PMG. *n* = 5 per group. (**B**) Primary microglia were treated with rmBACE1 protein with/without niclosamide treatment. Phosphorylated and total STAT3 were determined 12 h after treatment. *n* = 5 per group. (**C**, **D**) Quantification of pro- or anti-inflammatory cytokine gene expression is shown in the bar graphs, with data from at least 5 indicated treated PMGs. Data are shown as the relative change compared to the vehicle group. (**E**, **F**) *In vitro* analysis of the PMG erythrophagocytosis rate under rmBACE1 stimulation with/without niclosamide treatment, *n* = 3 per group. All data are presented as the mean ± SD. ^*^P < 0.05, ^**^P < 0.01.

To explore the mechanisms by which BACE-1 inhibits the beneficial phenotype of microglia, we noted that STAT3 was closely associated with the microglial polarization shift and microglia-dependent regulation of neuroinflammation and phagocytosis in stroke [13, 22]. In addition, STAT3 was reported to be regulated by BACE1. We assessed the expression levels of STAT3 and phosphorylated STAT3 in the *in vitro* ICH model after MK-8931 and vehicle treatment and found that Hb stimulation significantly promoted STAT3 phosphorylation, whereas MK-8931 treatment significantly reduced this alteration (Figure 5A). We therefore treated PMG with recombinant mouse BACE1 protein (rmBACE1) and/or the STAT3-specific inhibitor niclosamide. The results showed that BACE1 significantly promotes STAT3 phosphorylation, while this effect is blocked by niclosamide (Figure 5B). We further determined the role of STAT3 in PMG function. We indeed observed that BACE1 promoted the expression of a high level of proinflammatory genes in the PMG and that treatment with niclosamide effectively reduced the level of proinflammatory factor genes (IL-6, IL-1β, iNOS). In addition, niclosamide significantly upregulated the expression of anti-inflammatory (Arg-1, IL-10) and pro-phagocytic genes (CD36) (Figure 5C, 5D). Flow cytometry indicated that rmBACE1 strongly reduced the phagocytosis ratio of RBCs by PMG, but niclosamide treatment effectively reversed the inhibitory effect of rmBACE1 (Figure 5E, 5F). Finally, we overexpressed microglia STAT3 levels under MK-8931 treatment and the results showed that the regulation of both microglia inflammation and phagocytosis by MK-8931 was significantly blocked (Supplementary Figure 3A–3D). Thus, BACE1-mediated STAT3 activation modulates neuroinflammation and erythrophagocytosis of microglia.

### STAT3 inhibition ameliorated neurological impairment after ICH

**Figure 6:**
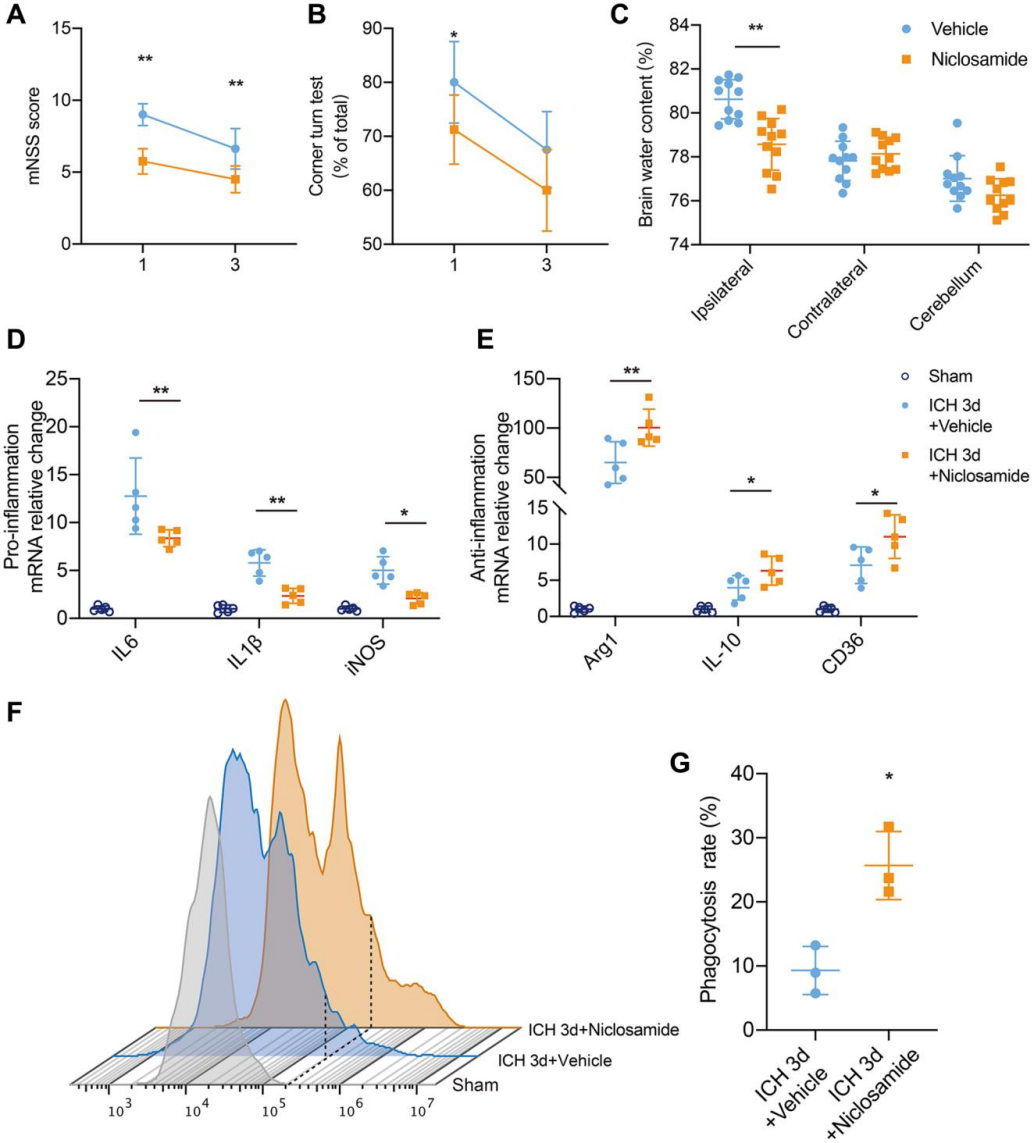
**STAT3 inhibition facilitated neurological recovery after ICH.** (**A**, **B**) Summarized results showing neurological assessment (mNSS score, corner-turning test) of MK-8931- or niclosamide-treated groups of mice at 1 and 3 days post-ICH. *n* = 8 per group. (**C**) Brain water content in groups of mice receiving the indicated treatments at Day 1 after ICH. *n* = 11 per group. (**D**, **E**) Analysis of the gene expression of inflammatory cytokines in sorted ICH 3-day microglia under the indicated treatment, *n* = 5 per group. Data are shown as the relative change compared to the sham group. (**F**) Flow cytometry histogram of *in vivo* RBC engulfment in microglia/macrophages. (**G**) Quantification of microglial phagocytosis in the indicated treated mice at Day 3 after ICH. *n* = 5 per group. All data are presented as the mean ± SD. ^*^P < 0.05, ^**^P < 0.01.

To investigate the effect of STAT3 inhibition on neurological function in ICH, we employed the corner turn test and mNSS score (Figure 6A). Administration of niclosamide resulted in improved neurological function and reduced brain water content at 1 day post ICH (Figure 6B, 6C). Similarly, the mNSS score also demonstrated that niclosamide significantly attenuated neurological impairments at both 1 and 3 days after ICH. We hypothesized that STAT3 inhibition would reduce BACE1-induced neuroinflammation by potentially impeding signal transduction activities. Specifically, microglia sorted from the mice with ICH 3 days after clonidine treatment showed a significant decrease in proinflammatory factor gene levels and a further increase in the expression of anti-inflammatory factor and pro-erythrophagocytosis molecule genes (Figure 6D, 6E). Finally, we used an *in vivo* RBC phagocytosis analysis system to determine the role of niclosamide in hematoma clearance. The results revealed that microglia of the mice with ICH 3 days under niclosamide treatment displayed a further elevated phagocytic ratio of involved erythrocytes (Figure 6F, 6G). In summary, inhibition of microglial STAT3 blocked the effect of BACE1 and had a positive effect on neuroprotection in ICH.

## DISCUSSION

This is the first study to demonstrate that microglial BACE1 is a key regulator of phagocytosis and the inflammatory phenotype after ICH. Importantly, we found that BACE1 is upregulated in microglia in the early phase of ICH, when there is abundant neuroinflammation, persistent hematoma compression, and blood-brain barrier disruption. We further revealed that the microglial phenotype in response to BACE1/STAT3 signaling accelerates inflammatory gene expression and cytokine secretion and inhibits the transition of the anti-inflammatory phenotype in parallel with diminished erythrophagocytosis. Pharmacological inhibition of BACE1 or STAT3 promotes hematoma resolution and modulation of inflammation, which further leads to recovery from neurological injury. Thus, this study suggests that microglial BACE1/STAT3-mediated phenotypic transition is a novel and vital mechanism involved in neurological injury after ICH and is a potential target for intervention.

Microglia are critical for cerebral homeostasis and injury recovery, and their phenotype has been recognized as an early and core indicator of progression in multiple hemorrhagic strokes [23, 24]. As a major defense against bleeding, the phagocytosis of RBCs and a proper inflammatory response define a beneficial microglial phenotype [19, 25, 26]. In addition, Microglia can also induce brain edema through blood-brain barrier disruption, and brain edema is often considered an adverse factor in neurological injury [27]. However, the exact reasons for the microglial shift toward an anti-inflammatory and pro-phagocytic phenotype during the early stages of ICH injury are largely unknown. Recently, BACE1 has been reported to be present in microglia, although it is widely considered a neuronal protein because it is robustly expressed in neurons [12]. In the present study, we demonstrated that BACE1 is expressed mainly by activated microglia and peaks at approximately 3 days. Importantly, BACE1 levels correlated with erythrophagocytosis and inflammatory gene expression *in vitro* and *in vivo*, consistent with previous findings obtained from LPS-induced inflammatory disorders and brain tumor microglia.

Several previous studies have reported that STAT3-deficient microglia upregulate IL-4-based anti-inflammatory cytokine expression after hemorrhagic stroke [22, 28, 29], which implies a protective phenotype against neuroinflammation. Moreover, microglia can be activated by IL-6, which is associated with the JAK-STAT3 pathway [30], and the STAT3 signaling pathway contributes to acute M1-related proinflammation, which may lead to early brain injury [31]. Furthermore, inhibition of the JAK-STAT3 pathway in a mouse model of AD abolished neuroinflammation by inhibiting microglial activation and upregulating BACE1 [32, 33]. In hepatocellular carcinoma. STAT3 inhibition facilitated the phagocytosis of tumor infiltrated DCs and macrophages (PMID:35665592). Meanwhile, STAT3 pathway was demonstrated to contribute to macrophage M2-polarization as well as phagocytosis by regulating the expressions of M1- and M2-related genes (PMID:35053345). Thus, we hypothesized that STAT3 is a downstream molecule of BACE1 and used *in vitro* models to confirm that inhibition of STAT3 phosphorylation effectively blocks BACE1-induced expression of proinflammatory factors, downregulation of anti-inflammatory factors, and impaired phagocytosis. Our *in vivo* data also demonstrate that inhibition of STAT3 promotes repair of nerve injury after ICH.

However, this study also has some limitations. The experimental animals in this study were all males, and the investigation of females was not included. Thus, the conclusions of this study are gender-limited, considering the influence of sexual factors on ICH [34]. The results of the *in vivo* part of this study are based on pharmacological inhibition of BACE1 and do not utilize knockout mice or AAV infection mediated BACE1 knockdown, which is one of the limitations of this study. In addition, due to the lack of *in vivo* BACE1 overexpression mouse models, in future studies, we will aim to generate a BACE1 overexpression or mutation mouse model to better validate its molecular mechanism as a therapeutic target after ICH and its relationship with the STAT3 signaling pathway.

In conclusion, the BACE1/STAT3 signaling pathway plays an essential role in mediating microglial phenotypes, which may be proinflammatory and diminish phagocytosis. Inhibition of either BACE1 or STAT3 alleviated adverse neuroinflammation and enhanced anti-inflammatory function and hematoma resolution in experimental ICH. These findings may provide us with a potential therapeutic approach aimed at modulating the microglial cell phenotype of ICH.

## MATERIALS AND METHODS

### ICH mouse model

All animal experiments were performed in accordance with the NIH Guide for the Care and Use of Laboratory Animals and were approved by the Animal Experimentation Ethics Committee of Shandong Provincial Hospital Affiliated with Shandong First Medical University. Male C57BL/6 mice (weight 25–28 g) were obtained from Shanghai Model Organisms (Shanghai, China). All animals were housed in pathogen-free conditions and had free access to food and water.

The ICH model was established in mice as described previously [21]. Mice were anesthetized with 3% isoflurane for induction and 1.5% isoflurane for maintenance. Tail was sterilized with 70% ethanol and then incised with a sterilized surgical blade. Next, autologous tail arterial blood was collected in a sterilized film. A total of 25 μL of whole autologous blood or the same volume of saline was collected by 50 μl Hamilton syringe injected into the striatum (0.5 mm anterior and 2.5 mm lateral to bregma, at a depth of 3.5 mm) at a rate of 2.5 μL/min after a midline scalp incision. The needle was withdrawn after a 5-minute pause. During surgery and anesthetic resuscitation, the animal’s body temperature was maintained 37 ± 0.5°C with an electric blanket.

### Administration of chemicals and regents

MK-8931 was purchased from MedChemExpress, USA (HY-16759). For *in vivo* experiments, MK-8931 was orally administered at a dose of 10 mg/kg per day until the mice were sacrificed [17]. For *in vitro* experiments, primary microglia treated with 100 nM MK-8931 [10] or vehicle were maintained in DMEM under 30 μM hemoglobin (Hb) exposure for 12 h [35].

Niclosamide was purchased from MedChemExpress (HY-B0497). For *in vivo* experiments, Niclosamide was intraperitoneally administered at a dose of 20 mg/kg per day until the mice were sacrificed [36]. For *in vitro* experiments, primary microglia treated with 5 μM Niclosamide or vehicle were maintained in DMEM under 5 ug/ml recombinant mouse BACE1 protein (R&D Systems, USA, 2976-AS) exposure for 12 h [37, 38].

#### Brain water content

Mice were sacrificed at 1d after ICH modeling and the brains were quickly removed and divided into the left hemisphere, right hemisphere, cerebellum, and brain stem. Each part was weighed immediately to get the wet weight. Then, the samples were dried at 105°C for 72 h to get the dry weight. The brain water content was calculated as ((wet weight − dry weight)/(wet weight)) × 100%.

### Behavioral assessment

Neurological dysfunction was assessed by a double-blind procedure on Days 1 and 3 after establishment of the ICH autologous blood model. The modified neurological severity score (mNSS) corner test was performed as previously described [21, 39]. Briefly, the mNSS score evaluates neurological function from a combination of motor, sensory, reflex and balance tests, with a score out of 15, with higher scores indicating more severe neurological deficits. In the corner test, mice are placed into a corner at an angle of 30° and required to turn to the left or right to exit the corner. Unilateral neurological impairment was determined by rating the percentage of right turns.

### Microglial isolation and fluorescence-activated cell sorting (FACS)

Single-cell suspensions of brain tissue were prepared as described previously [40, 41]. Briefly, mice were transcardially perfused with ice-cold medium A (HBSS, 15 mM HEPES, 0.05% glucose, and 1:500 DNase I) to remove blood cells after euthanized by isoflurane overdose. The tissue was mechanically ground with a Dounce homogenizer and then centrifuged at 340 g for 5 min. The supernatant was discarded, and the cells were resuspended in 30% Percoll (GE Healthcare, USA). The myelin sheath was then removed by centrifugation at 900 × g for 20 min. The sediment was collected and resuspended with CD45-AF700 (1:200; BioLegend, USA) Ly6G-BV421 (1:200; Biolegend), CD11b-PE/Cy7 (1:200; BioLegend) and Zombie NIR (1 μl/test; BioLegend) antibodies for 20 min at 4°C in the dark. All samples were analyzed with a CytoFLEXLX analyzer (Beckman, USA), or microglia was obtained by sorting with a BD Aria SORP system (BD, USA). All results were analyzed with FlowJo 10.8.1.

### Quantitative real-time PCR

Total RNA isolation (RNeasy Kit, Qiagen, USA) and cDNA reverse transcription were performed according to the manufacturer’s instructions (Vazyme, China). The primers were designed and provided by Tsingke Biotechnology Co., Ltd, China. Quantitative real-time PCR was performed according to the manufacturer’s instructions (Vazyme, China). The reaction conditions were as follows: first denaturation (95°C, 10 min), 40 denaturation cycles (95°C, 15 s), and extension (60°C, 40 s). Beta-actin was used as an internal control, and the relative levels of mRNA expression were calculated using the 2^−ΔΔCT^ formula. All data were shown as relative change to Sham or Vehicle group. The primers used for quantitative PCR are listed in Table 1 in the Appendix.

**Table 1:** Table of primer sequences.

**Gene**	**Sequence**
Il-6 forward	5′-GCCTTCTTGGGACTGATGCT-3′
Il-6 reverse	5′-TGTGACTCCAGCTTATCTCTTGG-3′
Tnfα forward	5′-ACCCTCACACTCACAAACCA-3′
Tnfα reverse	5′-ACCCTGAGCCATAATCCCCT-3′
Il-1β forward	5′-CAACCAACAAGTGATATTCTCCATG-3′
Il-1β reverse	5′-GATCCACACTCTCCAGCTGCA-3′
Ccl5 forward	5′-CCAATCTTGCAGTCGTGTTTGT-3′
Ccl5 reverse	5′-CATCTCCAAATAGTTGATGTATTCTTGAAC-3′
Inos forward	5′-CAACAGGGAGAAAGCGCAAA-3′
Inos reverse	5′-TGATGGACCCCAAGCAAGAC-3′
β-actin forward	5′-GAGACCTTCAACACCCCAGC-3′
β-actin reverse	5′-CCACAGGATTCCATACCCAA-3′
Cd163 forward	5′-TGCTGTCACTAACGCTCCTG-3′
Cd163 reverse	5′-CATTGCATGCCAGGTCATCG-3′
Cd206 forward	5′-GTCAGAACAGACTGCGTGGA-3′
Cd206 reverse	5′-AGGGATCGCCTGTTTTCCAG-3′
Il-10 forward	5′-GCTCCAAGACCAAGGTGTCT-3′
Il-10 reverse	5′-AGGACACCATAGCAAAGGGC-3′
Cd36 forward	5′-TCCTCTGACATTTGCAGGTCTATC-3′
Cd36 reverse	5′-AAAGGCATTGGCTGGAAGAA-3′
Arg1 forward	5′-AGCACTGAGGAAAGCTGGTC-3′
Arg1 reverse	5′-TACGTCTCGCAAGCCAATGT-3′

#### Hematoma measurement

Residual hematoma volume assessment was accessed based on the previously described methods [16]. Briefly, mouse brains were removed and cut into 1 mm thick coronal sections after cold PBS transcranial perfusion. The digital images of hematoma were acquired and analyzed with ImageJ software to obtain the residual hematoma area per section as well as the gray value. The residual hematoma volume was calculated: V = ∑(Areas of hematoma × 1). Similarly, the hematoma index was integrated by adding the difference in the gray value.

#### Primary microglia culture

P0–P1 C57BL/6 mice brains were dissected, and isolated cortices were isolated in cold HBSS and then dissociated with 0.25% trypsin for 12 min. The cell suspension was collected by DMEM containing 10% fetal bovine serum (FBS) then plated onto poly d-lysine-coated T75 cell culture flasks. 7 days later, 25 ng/ml GM-CSF was added into the culture system to accelerate microglial proliferation. Primary microglia were harvested by thermostatic shaking flasks at 180 rpm for 10 min. For further experiments, microglia were sustained in 3% FBS supplemented DMEM.

### RBC phagocytosis *in vivo*

Mice whole blood was collected by cardiac puncture after anesthetized. Whole blood was washed twice in PBS and then RBCs were isolated from the pellet after Ficoll gradient centrifugation in SepMate tubes (STEMCELL Technologies). Isolated RBCs were treated with a fluorescently labeled probe (PKH-26 Red Fluorescent Cell Linker Kit, Sigma-Aldrich, USA) or CellTracker CFSE dye (Invitrogen, USA) to generate fluorescent RBCs, and fluorescently labeled RBCs were mixed with plasma from the same donor mouse source. Then, 25 μl of reconstituted blood was injected into the basal ganglia of drug-pretreated mice in the same manner as the autologous injection ICH mouse model. After cold PBS perfusion, we carefully separated the tissue from the border of the hematoma with microsurgical forceps at a distance of 1 mm under the microscope. The phagocytosis of erythrocytes by microglia (identified as Zombie-Ly6G-CD45intCD11b+PKH26+) in the area surrounding the hematoma was analyzed by flow cytometry at 3 days post-ICH [42].

### RBC phagocytosis *in vitro*

The cell culture system was established as described previously [42]. Fluorescently labeled erythrocytes were added to primary microglia and incubated for 2 h at 37°C. Erythrocyte phagocytosis was observed using a STELLARIS 5 confocal microscope (Leica). The phagocytosis rate was calculated as the ratio of the number of microglia that phagocytosed erythrocytes to the total number of cells and is shown as a relative change from the control group.

### ELISA

Cell culture media were collected, and the secreted interleukin-10 (IL-10) and interleukin-1β (IL-1β) protein concentrations were measured by ELISAs. The culture medium was centrifuged at 4°C for 15 min at 12,000 g to collect the supernatant, which was then assayed and quantified with IL-10 and IL-1β ELISA kits (R&D Systems, USA) according to the manufacturer’s instructions.

### Immunofluorescence staining

Mice were euthanized and perfused with 4% paraformaldehyde (PFA), and whole brains were carefully removed and fixed by immersion in PFA, followed by dehydration with 30% sucrose. The brains were OCT-embedded to obtain 8-μm-thick frozen sections. For the staining procedure, brain slices were incubated in a blocking solution (10% donkey serum, 0.3% Triton 100 in PBS solution) at room temperature for 1 h. After PBS washes, the sections were incubated with primary antibodies overnight at 4°C. The following primary antibodies were used: Iba-1 (ab283346; Abcam) and BACE1 (ab183612; Abcam). After PBS washes, sections were incubated with fluorescent secondary antibody antibodies: donkey anti-rat Alexa Fluor 488 (A48269TR; Invitrogen) and donkey anti-rabbit Alexa Fluor 594 (A32754; Invitrogen). Finally, the brain slices were washed 3 times with PBS and then incubated with DAPI Fluoroshield mounting medium in the dark, followed by photography using confocal microscopy. All the images were taken at the peri-hematoma area.

### Western blot

The standard protocol was performed as previously described [21]. Briefly, cells were lysed on ice for 20 min with RIPA buffer containing protease inhibitor and phosphatase inhibitor. After buffer was loaded, all samples were subjected to SDS-PAGE and blotted onto PVDF membranes. After blocking with 5% fetal bovine serum (BSA) in TBST, membranes were incubated with primary antibodies overnight at 4°C. The primary antibodies were rabbit-anti-BACE1 antibody (ab183612, Abcam), rabbit-anti-STAT3 antibody (A19566, Abclonal), and rabbit-anti-phospho-STAT3-S727 antibody (ab267373, Abcam). After three washes with TBST, the membranes were incubated with HRP-conjugated anti-rabbit secondary antibodies in 5% BSA for 2 hours at room temperature. The signals on the membranes were developed with ECL HRP substrate, and images were acquired with a molecular imager (ChemiDoc MP, Bio-Rad) and analyzed with ImageJ.

#### siRNA transfection

Primary microglia plated in 24-well plates (70% confluent) were transfected with siRNA for the mouse BACE1 gene or negative control using Lipofectamine RNAiMAX according to the manufacturer’s protocol. Briefly, 10 pmoles of siRNA and 4.5 μL of the transfection reagent were mixed in 50 μL of OPTI-MEM for 10 min at room temperature. The siRNA complex suspension was added dropwise to the 24-well cell culture plate. The siRNA was designed and synthesized in the company of Tsingke Biotechnology, China. The gene sequences were designed as follows: BACE1-siRNA (5′ to 3′): Forward-GCUUUGUGGAGAUGGUGGATT, Reverse-UCCACCAUCUCCACAAAGCTT; The negative control (NC) siRNA (5′ to 3′): Forward-GCUUUGUGGAGAUGGUGGATT, Reverse-UCCACCAUCUCCACAAAGCTT.

#### Plasmids transfection

Polyethylenimine (PEI) was used to transfect primary microglia post-isolation. STAT3 sequences (NM_011486.5) were constructed in the pcDNA3.1 vector. Briefly, PEI (1 mg/ml) was added to OPTI-MEM with desired amount of DNA (1.5:1 PEI:DNA) and allowed to incubate at room temperature for 15 min. The PEI/DNA transfection mixture was then added directly onto cells and allowed to incubate for 2 h. Cells were then washed and incubated in completed media (3%FBS in DMEM) for 72 h after transfection.

### Statistics

All data are shown as the mean ± SD. Statistical differences between two groups were tested by two-tailed Student’s *t* test or Wilcoxon’s signed-rank test, and one-way ANOVA was used to compare multiple groups. Two-way ANOVA accompanied by a Bonferroni post hoc test was used for multiple comparisons. Significance was defined as *P* < 0.05. All results were analyzed by investigators blinded to the experimental group using GraphPad Prism8 software.

## Supplementary Materials

Supplementary Figures
