# Supplementary material for: Inhibition of BACE1 attenuates microglia-induced neuroinflammation after intracerebral hemorrhage by suppressing STAT3 activation
Source: Aging (Albany NY). 2023 Aug 7;15(15):7709–26. doi: 10.18632/aging.204935 (PMC10457076; doi:10.18632/aging.204935)
Supplement: Supplementary Figures [file aging-15-204935-s001.pdf]

SUPPLEMENTARY FIGURES

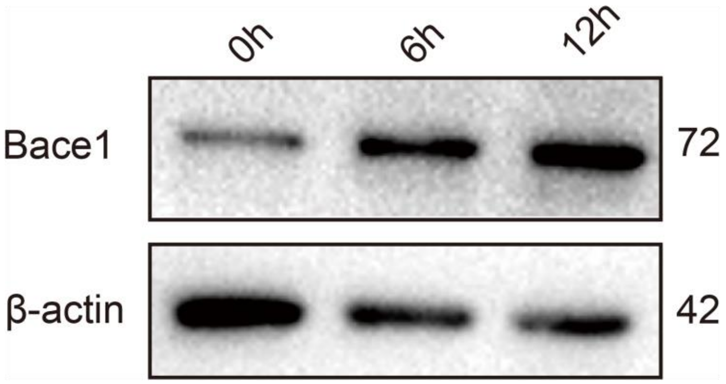

Supplementary Figure 1. Representative blot images of BACE1 protein levels in primary cultured mouse microglia post-hemoglobin stimulation.

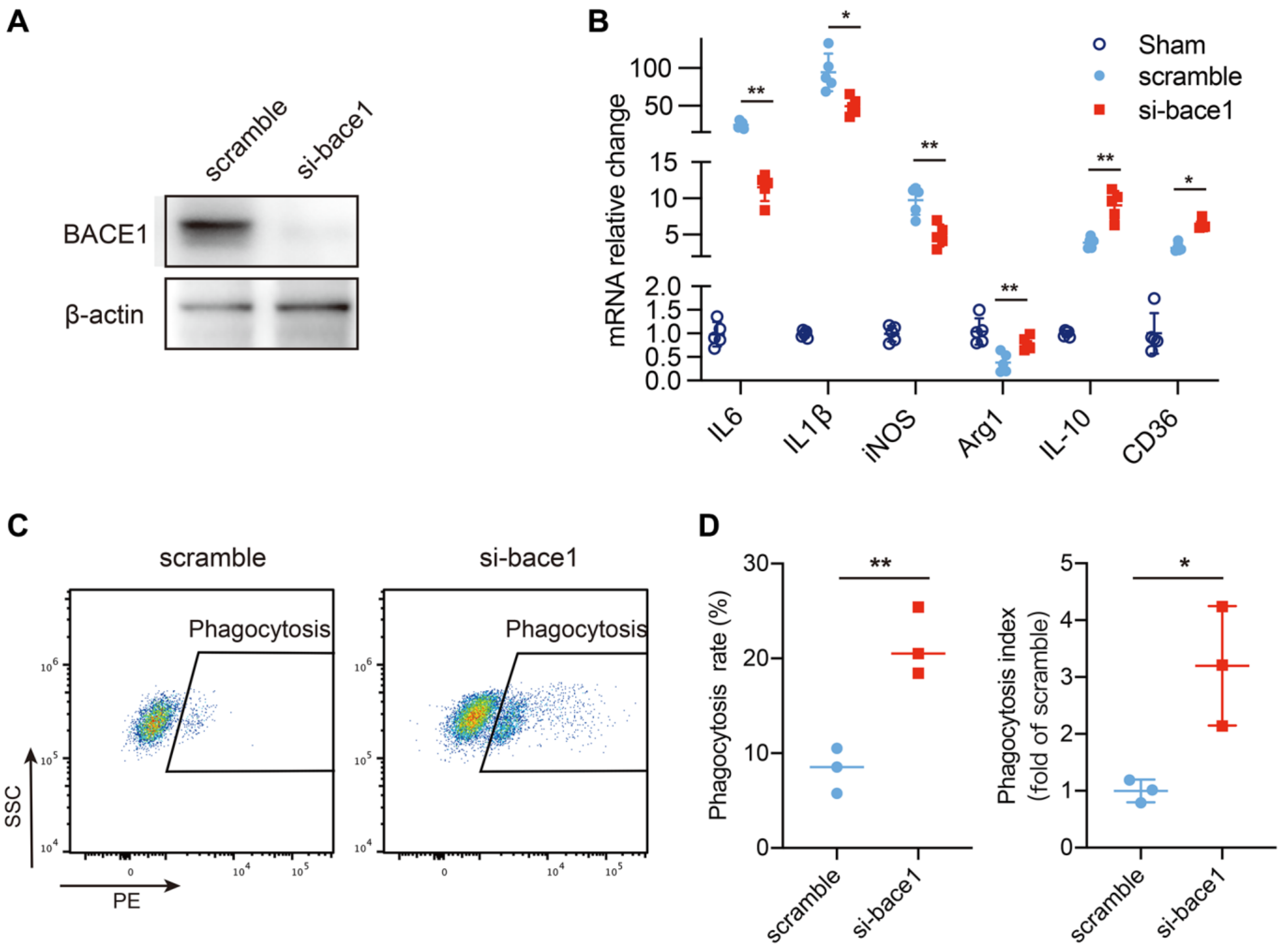

Supplementary Figure 2. BACE1 induced pro-inflammatory microglia and impaired the phagocytosis *in vitro*. *In vitro* ICH model was induced in PMG by 12 h hemoglobin stimulation. (**A**) Confirmation of siRNA-targeted Bace1 transfection by immunoblotting. (**B**) Genes expression is shown in the bar graphs, with data from at least 5 independent experiments. Data are shown as relative change of Sham group, $^{*}P < 0.05$ and $^{**}P < 0.01$. (**C**, **D**) *In-vitro* analysis of PMG erythrophagocytosis by flowcytometry. All Data are presented as mean ± SD. $^{*}p < 0.05$, $^{**}p < 0.01$.

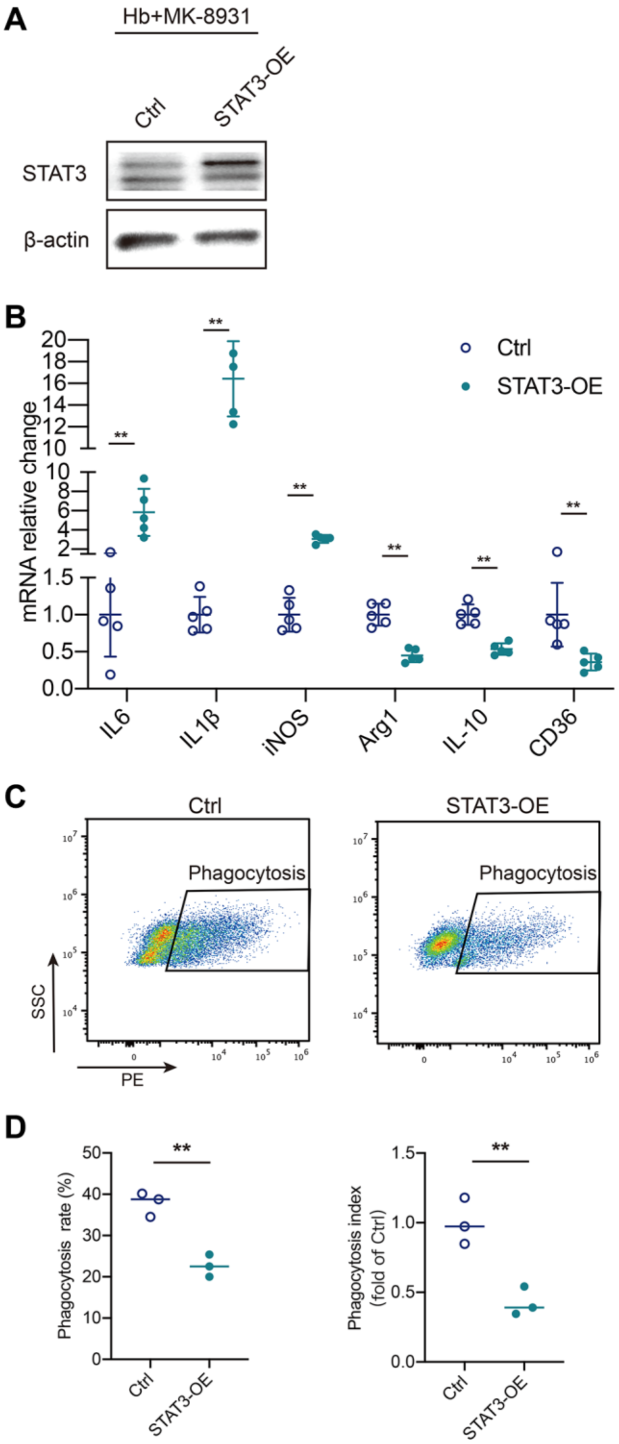

Supplementary Figure 3. STAT3 rescue diminished the effects of MK-8931 in PMG. (**A**) The validation of STAT3 overexpression in PMG by immunoblotting. (**B**) STAT3 overexpressed microglia showed higher pro-inflammatory genes mRNA levels while impaired the anti-inflammatory and pro-phagocytosis genes expression under MK-8931 treatment. (**C**, **D**) *In-vitro* analysis of PMG erythrophagocytosis by flowcytometry. STAT3 overexpression blocked the BACE1 inhibitor effect. All Data are presented as mean ± SD. $^{**}p < 0.01$.
